# Metabolic syndrome is linked to a mild elevation in liver aminotransferases in diabetic patients with undetectable non-alcoholic fatty liver disease by ultrasound

**DOI:** 10.1186/1758-5996-2-65

**Published:** 2010-11-03

**Authors:** Alireza Esteghamati, Arsia Jamali, Omid Khalilzadeh, Sina Noshad, Mohammad Khalili, Ali Zandieh, Afsaneh Morteza, Manouchehr Nakhjavani

**Affiliations:** 1 Endocrinology and Metabolism Research Center (EMRC), Vali-Asr Hospital, School of Medicine, Tehran University of Medical Sciences, Tehran, Iran; 2 Advanced Diagnostic and Interventional Radiology Research Center (ADIR), Imaging Medical Center, Imam Hospital, Tehran University of Medical Sciences, Tehran, Iran

## Abstract

**Background:**

Despite ongoing findings on the relationship between elevated levels of alanine and aspartate aminotransferases (ALT and AST) and metabolic syndrome (MetS), this association in diabetic patients without a known cause for liver enzymes elevation other than diabetes, per se, remains unclear. In this study, we aimed to assess the relationship between circulating liver enzymes and MetS in a relatively large sample of patients with diabetes.

**Methods:**

A total of 670 diabetic patients, without known causes of hepatocellular injury, were enrolled. Patients with ultrasonographic signs of fatty liver disease were not included. Fasting blood samples were obtained and biochemical characteristics were measured. MetS was defined according to the international diabetes federation criteria.

**Results:**

Serum ALT and AST were significantly higher in patients with MetS (p < 0.001). High waist circumference and low HDL-cholesterol were significantly associated with elevated ALT (OR = 2.56 and 2.0, respectively) and AST (OR = 2.23 and 2.21, respectively). ALT and AST were significantly associated with MetS (OR = 2.17 and 2.31, respectively). These associations remained significant after multiple adjustments for age, sex, BMI, diabetes duration, HbA1c and medications. There was a significant (p < 0.01) positive association between the number of the MetS features and the level of ALT or AST.

**Conclusion:**

In diabetic patients without ultrasonographic evidence of fatty liver, elevated aminotransferases are independently associated with MetS. Despite negative ultrasound results in diabetic patients with MetS, the serum level of liver aminotransferases may be elevated and should be more thoroughly monitored.

## Introduction

Insulin insensitivity is a known cause of liver damage [1]. Elevation of circulating liver enzymes including aspartate aminotransferase (AST), alanine aminotransferase (ALT), and alkaline phosphatase (ALP) is suggestive of hepatocellular injury [2-5]. There is increasing evidence that ALT is significantly and independently associated with type 2 diabetes mellitus [6-8], however not all of the studies support this finding [9].

In recent years, nonalcoholic fatty liver disease (NAFLD), as a novel component of insulin resistance and metabolic syndrome (MetS), has drawn the attention of many researchers. NAFLD encompasses a wide spectrum of liver diseases ranging from simple benign steatosis to steatohepatitis, fibrosis, and cirrhosis [1]. This condition which is associated with long-standing elevations in liver enzymes [10,11], is related to higher risk of adverse cardiovascular events, oxidative stress, endothelial dysfunction, and MetS [12].

Despite ongoing findings on the relationship between NAFLD and MetS [13], the relationship between elevated liver enzymes and MetS in diabetic patients without a known cause for liver enzymes elevation other than diabetes, per se, remains unclear. In particular, while most of the studies describe the association between MetS and elevated liver aminotransferases via the NAFLD mechanism [14]; it is not clear what extent of liver steatosis is sufficient to mediate the association between liver enzymes and MetS in diabetes. To best of our knowledge, no evidence is available regarding the association between MetS and serum aminotransferases in patients with mild stages of liver steatosis. To determine subjects with mild liver steatosis from those with advanced stages, ultrasonography is an appropriate screening tool. Ultrasonography with the sensitivity of 60-89 percent and specificity of 66-93 percent in detecting steatosis is proved as a good tool for detection of clinically significant fatty infiltrations, in epidemiologic studies [15]. It is reported that individuals with negative fatty liver changes in ultrasonography have hepatic fat <30% [16].

Of note, there is limited evidence regarding the pattern of abnormality in liver enzymes in diabetic patients with and without MetS. In this study, we aimed to assess the relationship between circulating liver enzymes and MetS in a relatively large sample of Iranian patients with type 2 diabetes, after excluding patients with ultrasonographic signs of NAFLD or any other known causes of hepatocellular injury.

## Methods

### Study population

The study population consisted of 670 diabetic subjects who consecutively visited Vali-Asr hospital outpatient diabetes clinic (Tehran, Iran) from June 2007 to September 2009. Diabetes was diagnosed according to American Diabetes Association (ADA) criteria [17]. The study population was divided into two groups of diabetic patients with (n = 502) and without MetS (n = 168). MetS was defined according to the IDF criteria using the cutoffs we recently established for waist circumference (WC) in Iranian adults [18]. Subjects with abdominal obesity (WC > 90 cm for both men and women) plus at least two of the risk factors from the IDF criteria including high triglyceride levels (TG ≥ 150 mg/dL), low HDL-cholesterol (HDL-C ≤ 40 mg/dL in men and ≤ 50 mg/dL in women), high blood pressure (systolic BP ≥ 130 mmHg, diastolic BP ≥ 85 mmHg or treatment of previously diagnosed hypertension), and high fasting plasma glucose level (FPG ≥ 100 mg/dL or treatment of previously diagnosed diabetes) were considered to have MetS. Since all of our patients were known cases of diabetes, abdominal obesity plus at least one of the above factors was sufficient to establish a clinical diagnosis of MetS in this study.

A thorough history was taken and physical examination was performed by a trained physician for all patients. None of our patients were on insulin or thiazolidinedione treatment. Patients with a history of alcohol consumption, as well as those with history of a known chronic liver disease including autoimmune hepatitis, hemochromatosis, Wilson's disease, primary biliary cirrhosis, and sclerosing cholangitis were not included in the study. No subject had history of taking medications that are commonly attributed to liver enzyme elevations (such as methotrexate, tetracycline, amiodarone, high doses of estrogen, tamoxifen, diclofenac, amoxicillin, valproic acid or steroids). Abdominal ultrasonography for evaluation of liver (Hitachi EUB 405 apparatus equipped with a convex 3.5 MHz probe) was performed in patients, by a single experienced radiologist (to avoid inter-operator discordance) and those with evidence of fatty liver were excluded. Biochemical evaluation including serum ALT and AST was performed in all subjects. All patients with elevated liver enzymes underwent serologic examination for hepatitis B and C viruses, and those with positive results were not included in the study. Auto antibodies (antinuclear antibody and anti-smooth muscle antibody) and biochemical features (including iron studies, ceruloplasmin, and urinary copper) were also measured in patients with elevated liver enzymes and those with abnormal results were not included.

The study was approved by the local ethics committee of Tehran University of Medical Sciences. All subjects were provided with written informed consent and the study was conducted in accordance with Helsinki declaration.

### Data collection

Anthropometric data including age, sex, height, weight and WC were collected. Height was measured with subject standing without shoes by a standard stadiometer and the nearest one centimeter was recorded. Weight was measured with subjects wearing only light clothing standing on a digital scale; the nearest 0.5 kilogram was recorded. WC was measured at the end of a normal expiration, midway between the inferior margin of the ribs and superior border of the iliac crest in a horizontal plane, and was rounded to the nearest 0.1 cm, according to the previously published literature [19]. Body mass index (BMI; kg/m^2^) was calculated according to the Quetelet equation (weight in kilograms divided by height in square meter) [20]. BP was measured on the right arm with subject having relaxed for at least 5 minutes, via a standard sphygmomanometer of appropriate cuff size and was repeated with 10 minutes interval. The average of these two measurements was recorded for systolic and diastolic BP.

### Laboratory methods

Venous blood samples were collected following an overnight 12-hours of fasting and HbA1c, TG, cholesterol (Chol), HDL-C, low density lipoprotein-cholesterol (LDL-C), fasting plasma glucose (FPG), AST, ALT and ALP were measured. FPG measurement was carried out using the glucose oxidase method. Chol, TG, LDL-C, and HDL-C were determined using enzymatic methods. Analyses of serum ALT, AST and ALP were performed using enzymatic photometry. ALT level > 30 U/L in women and > 40 U/L in men, AST level > 30 U/L in women and > 36 U/L in men and ALP levels of greater than 306 U/L in both women and men were considered elevated according to the manufacturer's instructions. Serum ALT and AST were measured by the IFCC (International Federation of Clinical Chemistry and Laboratory Medicine) method (ALT intra-assay coefficient of variation [CV] = 3.7%, AST intra-assay CV = 2.5%). Serum ALP was measured using the DGKC (Deutsche Gesellschaft für Klinische Chemie) method (Intra-assay CV = 1.5%). These measurements were conducted using commercial Parsazmun kits (Tehran, Iran) and a Hitachi 704 automatic analyzer (Tokyo, Japan) [21]. HbA1c was measured using the high performance liquid chromatography method. For diagnosis of hepatitis B and C, antibodies to hepatitis C virus, hepatitis B surface antigen, hepatitis B surface antibody, hepatitis B core antibody, hepatitis B e antigen, and hepatitis B e antibody were measured using commercially available enzyme linked immunosorbent assay (ELISA) kits (DRG Diagnostics GmbH, Germany).

### Statistical analysis

All statistical analyses were performed using SPSS software version 17.0 for windows (SPSS Inc. Chicago, IL, USA). Continuous variables are expressed as mean ± standard deviation. Independent sample t-test was used to identify differences of continuous variables between groups with and without MetS. Categorical variables were compared using Chi square analysis. The degree of association between continuous variables was assessed via Pearson's correlation coefficient (r). Logistic regression analysis was performed to assess the independent correlations between different features of MetS, and ALT or AST. General linear models were used to compare the adjusted mean values of ALT or AST in patients with different numbers of metabolic abnormalities. P value < 0.05 was considered statistically significant.

## Results

A total of 670 diabetic patients without ultrasound evidence of fatty liver, with the mean age of 54.12 ± 10.49 years (range: 18-80 years) were included in the study. Females constitute 360 (53.7%) of the patients. The mean and median of diabetes duration in the study population was 5.84 ± 5.69 and 5.00 years, respectively. The prevalence of MetS in the patients was 74.9%. About 31.5% of the patients (n = 204) had elevated aminotransferases, 110 had only elevated ALT, 13 had only elevated AST and the remaining 81 patients had both elevated ALT and AST. Elevation in ALT or AST was mild in all our patients (less than 2.5 times elevation compared with upper limit of normal range).

As Table 1 summarizes, there were no significant differences in the age, sex, diabetes duration, HbA1c, FPG, LDL-C, Chol, and ALP levels in the patients with and without MetS. However, BMI, WC, diastolic and systolic BP, TG, HDL-C, AST and ALT levels were higher in patients with MetS. Furthermore, there were no significant differences between patients with and without MetS with respect to use of oral anti-diabetic medications, lipid lowering or antihypertensive agents. Also there were no significant association between elevated ALT/AST and use of oral anti-diabetic medications (P = 0.589 and P = 0.224 for elevated ALT and elevated AST, respectively), lipid lowering agents (P = 0.859 and P = 0.262, respectively) or anti-hypertensive medications (P = 0.718 and 0.181, respectively). Both long term control of glycemic situation measured by HbA1c level, and short term glycemic control quantified by FPG was similar in patients with and without elevated ALT (P = 0.20 and 0.69, respectively).

**Table 1:** Clinical and laboratory characteristics of the study subjects with and without metabolic syndrome (MetS).

Variables	Without MetS	With MetS	*P *value
N (%)	168 (26.6)	502 (74.9)	-
Age (years)	53.39 ± 10.34	54.36 ± 10.54	0.300
Females (n, %)	101 (60.11)	259 (51.59)	0.054
Diabetes duration (years)	5.94 ± 6.35	5.81 ± 5.46	0.800
Body mass index (kg/m^2^)	25.96 ± 3.48	30.78 ± 4.69	< 0.001*
Waist circumference (cm)	88.59 ± 8.43	102.94 ± 8.67	< 0.001*
Diastolic blood pressure (mmHg)	78.77 ± 7.28	81.56 ± 8.19	< 0.001*
Systolic blood pressure (mmHg)	122.50 ± 13.92	130.94 ± 17.34	< 0.001*
Anti hypertensive medications ^a ^(n, %)	68 (40.50)	200 (39.84)	0.917
Anti-Diabetic Medications (n, %)			0.470
*No medication*	0 (0)	2 (0.01)	
*Glibenclamide*	29 (17.8)	71 (14.1)	
*Metformin*	34 (20.2)	123 (24.5)	
*Glibenclamide & Metformin*	63 (37.5)	206 (41.0)	
Lipid Lowering Agents (n, %)			0.189
*No medication*	103 (61.31)	271 (53.98)	
*Statins*	53 (31.55)	174 (34.66)	
*Fibrates*	4 (2.38)	30 (5.97)	
*Statins & Fibrates*	8 (4.76)	22 (4.38)	
HbA1c (%)	7.68 ± 1.92	8.03 ± 1.77	0.130
Fasting plasma glucose (mg/dL)	162.86 ± 58.13	166.49 ± 53.23	0.445
Triglycerides (mg/dL)	158.21 ± 90.03	213.01 ± 118.73	< 0.001*
HDL-C (mg/dL)	48.16 ± 11.26	44.23 ± 12.60	< 0.001*
LDL-C (mg/dL)	115.59 ± 37.77	111.73 ± 36.05	0.236
Total cholesterol (mg/dL)	195.08 ± 44.84	197.29 ± 45.34	0.583
Alanine aminotransferase (U/L)	27.30 ± 15.23	31.76 ± 18.74	0.006*
Aspartate aminotransferase (U/L)	21.53 ± 9.58	24.17 ± 13.84	0.007*
Alkaline phosphatase (U/L)	148.44 ± 92.83	151.95 ± 77.01	0.653

*P value < 0.05

Variables are presented as mean ± standard deviation unless stated otherwise.

^a ^Medication used for treatment of hypertension include angiotensin converting enzyme inhibitors, angiotensin receptor blockers, calcium channel blockers and beta blockers.

In univariate analysis, serum ALT level was significantly (p < 0.05) correlated with AST (r = 0.758), age (r = 0.196), diabetes duration (r = 0.149), WC (r = 0.200), TG (r = 0.159) and was negatively associated with HDL-C (r = -0.165). No significant correlation was observed between ALT and ALP level, BP (systolic or diastolic), HbA1c, FPG, and LDL-C. Similar to ALT, AST was significantly (p < 0.05) correlated with age (r = 0.129), diabetes duration (r = 0.123), WC (r = 0.188), and was negatively associated with HDL-C (r = -0.137). No significant correlation was observed between AST and TG, ALP, BP (systolic or diastolic), HbA1c, FPG or LDL-C.

**Figure 1:**
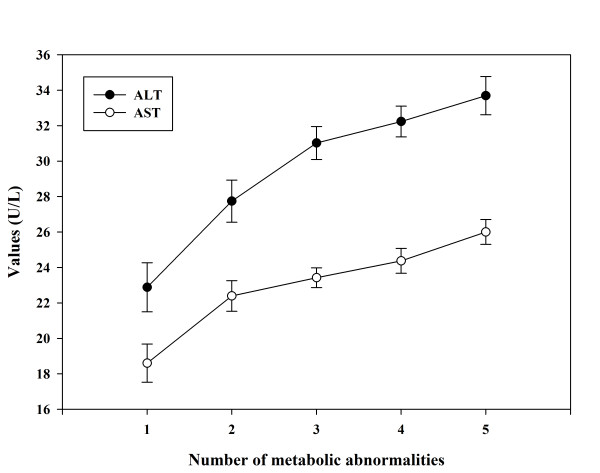
**There was a significant (p < 0.01) association between the number of the metabolic abnormalities and ALT or AST, after adjustment for age, sex and BMI, HbA1c, diabetes duration and medications**.

As shown in Table 2, patients with elevated ALT had significantly higher prevalence of impaired TG levels. However, after adjustment for various confounding factors the significant association disappeared. In addition, the prevalence of Low HDL-C and high WC was significantly higher in patients with elevated ALT and remained significant even after multiple adjustments (OR = 2.04, 95% CI: 1.24-3.38, and OR = 2.48, 95% CI: 1.20-5.11, respectively). Elevated levels of AST had no significant association with either high BP or high levels of serum TG. Furthermore, although patients with elevated AST had higher prevalence of low HDL-C and high WC, only HDL-C remained significant after adjustment for age, sex, BMI, diabetes duration, HbA1c, and various medications (OR = 2.13, 95% CI: 1.06-4.31). Both elevated ALT and AST had a significant (p < 0.01) association with increase in the number of features of MetS (Figure 1).

**Table 2:** Features of the metabolic syndrome in patients with and without elevated liver aminotransferases

	Diabetic patients			
			
Variables (n, %)	without increased ALT (n = 479)	with increased ALT (n = 191)	Crude OR (95% CI)	Adjusted OR (95% CI)
High blood pressure	203 (42.4)	91 (47.6)	1.24 (0.88-1.73)	1.11 (0.68-1.80) ^1^
High triglycerides	286 (59.7)	135 (70.7)	1.63 (1.13-2.33)*	1.50 (0.91-2.48) ^1^
Low HDL-C	256 (53.4)	133 (69.6)	2.00 (1.40-2.58)***	2.04 (1.24-3.38) **^1^
High waist circumference	368 (26.8)	171 (89.5)	2.56 (1.53-4.26)***	2.48 (1.20-5.11) *^1^
MetS	341 (71.2)	161 (84.3)	2.17 (1.40-3.36)***	2.08 (1.12-3.87) *^1^

	**without increased** **AST** **(n = 576)**	**with increased** **AST** **(n = 94)**		

High blood pressure	249 (43.2)	45 (47.9)	1.21 (0.78-1.87)	1.05 (0.56-1.98) ^2^
High triglycerides	354 (61.4)	67 (71.3)	1.56 (0.97-2.51)	1.39 (0.71-2.73) ^2^
Low HDL-C	320 (55.6)	69 (73.4)	2.21 (1.36-3.59)**	2.13 (1.06-4.31) *^2^
High waist circumference	405 (70.3)	84 (89.4)	2.23 (1.12-4.43)*	2.17 (0.89-5.31) ^2^
MetS	421 (73.1)	81 (86.2)	2.31 (1.25-4.27)**	2.23 (1.09-4.57) *^2^

*P < 0.05, **P < 0.01, ***P < 0.001

^1^Adjusted for age, sex, BMI, diabetes duration, HbA1c and medications (anti diabetic, lipid lowering or antihypertensive agents)

^2^Adjusted for age, sex, BMI, diabetes duration, HbA1c and medications (anti diabetic, lipid lowering or antihypertensive agents)

## Discussion

This study demonstrates that in a reasonably large sample of patients with diabetes, liver enzymes are significantly higher in patients with MetS, in spite of similar long and short-standing glycemic control indices and similar diabetes duration. We also observed that elevated liver enzymes are associated with age, diabetes duration and features of MetS including serum HDL-C level and WC. Oral anti-diabetic, anti-hypertensive, and lipid lowering agents could minimally influence our observed associations, since there was no significant difference between use of medication in patients with and without MetS and also in patients with elevated and normal liver enzymes.

There are very few studies assessing the association between liver enzymes and MetS in patients with diabetes. Hickman et al (2007), on a sample of 189 patients with diabetes, showed that MetS is associated with an unexplained increase in ALT, despite similar glycemic control in patients with and without MetS [22]. In this study, liver enzymes other than ALT including AST and ALP were not assessed. In another study by Forlani and collegues (2008), it was shown that presence of MetS is a significant predictor of raised ALT [23]. These studies did not evaluate the presence of NAFLD in the participants. In the present study, for the first time, we excluded cases with ultrasonographic signs of NAFLD, focusing on the patients with elevated liver enzymes without any known cause, except diabetes, per se. Our results showed an independent association between MetS and mildly elevated AST/ALT in diabetes patients without ultrasound evidence of fatty liver disease. This can be due to a mild underlying steatohepatitis, which is undetectable by ultrasound. Our results can be interpreted in parallel with the findings of the study by Mofrad et al (2003), which showed that even low normal ALT values can be associated with underlying steatohepatitis, especially in diabetic patients [24].

The pathogenesis of liver damage in patients with diabetes is not thoroughly understood and most of our current knowledge is related to the connection between NAFLD and insulin resistance [14,25]. Hepatic insulin resistance can play an important role in liver dysfunction and elevation of liver enzymes [8,26]. Also, inflammatory cytokines including tumor necrosis factor and interleukin 6 are proposed in pathogenesis of hepatocellular injury in insulin resistance/MetS [14]. Increased levels of TG and free fatty acids causes accumulation of TG in nonadipose tissues including liver [27] which results in lipid peroxidation. Subsequently, mitochondrial dysfunction caused by formation of toxic metabolites and reactive oxygen species induces cell apoptosis [14,25,28]. The association between MetS and liver enzymes in our study shows that in diabetes, MetS is possibly linked to undetectable mild stage of NAFLD. This notifies clinicians to more thoroughly monitor serum aminotransferase in diabetic patients, particularly when the diabetic patient has MetS as well. NAFLD is a feature of MetS [29], and in diabetic patients with MetS, regardless of negative ultrasound results, NAFLD might be present and contribute to the complex of different features of MetS.

This study had potential limitations: first, the cross-sectional nature of this study precludes cause and effect relationships. Therefore, further longitudinal studies are paramount to investigate the role of liver enzymes as risk factors of the MetS. Second, it should be noted that liver biopsy is considered as the gold standard of diagnosis of NAFLD [30,31], however it is invasive, expensive, and may bear potential risks [32]. There are some imaging techniques for assessing hepatic fat content. Non-enhanced Computed Tomography (CT) can qualitatively detect macrovesicular steatosis of 30% and more, however there are conflicting evidence on its value in quantifying liver fat content [32]. Enhanced CT is less valuable in assessing liver fat since contrast type, injection rate, and timing can significantly influence the liver to spleen attenuation difference for diagnosing fatty liver [33,34]. Several studies have demonstrated an appropriate correlation between severity of steatosis on magnetic resonance imaging (MRI) and histologic biopsies [35-37]. Emerging imaging techniques such as dual gradient echo MRI (DGE-MRI) and proton magnetic resonance spectroscopy (^1^H-MRS), are currently evaluated for detecting hepatic steatosis. One study has demonstrated DEG-MRI is highly accurate for evaluating moderate to severe hepatic steatosis (sensitivity and specificity of greater than 90%) and fairly sensitive (sensitivity = 76.7%) and specific (specificity = 87.1%) for detection of all degrees of steatosis [38]. According to our current knowledge, most studies have shown that ^1^H-MRS is the method of choice for detecting steatosis, with a high correlation between the fat fraction estimated by this method (r = 0.70 and 0.71) and histologic evaluation [38-40]. Nevertheless, we believe that for the purpose of this epidemiologic study, ultrasonography is an appropriate screening tool for determining negative or mild stages of fatty liver disease, with a reasonable accuracy [15]. Our study provided significant insight to our knowledge on the association between elevated liver enzymes and MetS in diabetic patients with mild stages of fatty liver, undetectable in ultrasound. An interesting topic, which needs further attention in future studies, is the significance of serum gamma-glutamyl transpeptidase (GTP) in diabetic patients, and its association with MetS. It is interesting to compare GTP with AST or ALT in prediction of MetS in a diabetes status.

In conclusion, we showed an independent association between elevated ALT/AST and MetS in diabetic patients with undetectable mild stages of NAFLD. Despite negative ultrasound results, the serum level of liver aminotransferases may be elevated in diabetic patients with MetS and should be more thoroughly monitored.

## Conflict of interests

The authors declare that they have no competing interests.

## Authors' contributions

AE conceived the study, participated in its design, coordination and acquisition of data. AJ wrote the first draft and contributed to the statistical analysis. OK participated in manuscript writing, statistical analysis and acquisition of data. SN and MK helped in drafting the manuscript, statistical analysis and interpretation of the results. AZ participated in acquisition of data, writing and editing the manuscript. AM helped to perform the statistical analysis and participated in writing. MN participated in interpretation of the results and editing the manuscript. All authors read and approved the final manuscript.
